# Incidence, ecological factors, and temporal trends of prison homicides and suicides in Chile, 2000-2024

**DOI:** 10.3389/fpsyt.2025.1715635

**Published:** 2026-01-22

**Authors:** Benjamín Asencio-Rojas, Gabriel Cavada-Chacón, Enzo Rozas-Serri, Adrian P. Mundt

**Affiliations:** 1 Programa de Doctorado en Salud Pública, Instituto de Salud Poblacional, Facultad de Medicina, Universidad de Chile, Santiago, Chile; 2 Programa de Magíster en Salud Pública, Instituto de Salud Poblacional, Facultad de Medicina, Universidad de Chile, Santiago, Chile; 3 Programa de Epidemiología, Instituto de Salud Poblacional, Facultad de Medicina, Universidad de Chile, Santiago, Chile; 4 Departamento de Psiquiatría y Salud Mental, Hospital Clínico Universidad de Chile, Santiago, Chile; 5 Departamento de Neurología y Psiquiatría, Clínica Alemana de Santiago, Facultad de Medicina Clínica Alemana, Universidad del Desarrollo, Santiago, Chile; 6 Centro de Investigación Biomédica, Facultad de Medicina, Universidad Diego Portales, Santiago, Chile

**Keywords:** Chile, external causes of death, homicide, prison, suicide

## Abstract

**Aims:**

External (non-natural) causes of death are frequent in prison. This study aimed to assess the incidence, associated ecological factors, and changes over time of prison homicides and suicides in Chile between 2000 and 2024.

**Methods:**

National and regional cases of homicides and suicides in prison were obtained by year through Transparency Council requests to the Chilean prison authority. Corresponding incidence data in the general population were retrieved from publicly available sources. Incidence rates were estimated per 100,000 person-years of imprisonment and compared between regions. The proportion of external causes of death among overall mortality in prison was also calculated. Prais-Winsten autoregressive models were used to analyze changes in incidence over time. Latent structures underlying the incidence were explored using principal component analysis (PCA).

**Results:**

Incidence rates of external causes of death were 148 (95% CI: 127-170) in southern, 142 (95% CI: 133-151) in central, and 86 (95% CI: 74-99) per 100,000 person-years in northern regions. A decreasing slope in the national homicide incidence was observed (ß=–12.26, p<0.0001), while several regions presented an increase (i.e., Los Ríos, a southern region; ß=21.57, p<0.0001). Suicide incidence remained stable over time (ß=0.44, p=0.385). Settings with higher proportions of individuals convicted of violent offences and southern regions faced an elevated risk of suicide (ß=18.63, p=0.007).

**Conclusions:**

Prevention policies and interventions for external causes of death should prioritize southern regions, those with increasing incident rates and with high proportions of individuals sentenced for violent offences.

## Introduction

The correctional system of Chile houses more than 60,000 persons in overcrowded facilities, operating at 139% of its capacity. Occupancy has increased in recent years (1–3). In 2024, the country reported an incarceration rate of approximately 300 per 100,000 population. About 40% of the imprisoned individuals were on remand. Turnover is high, with a higher number than the entire population being newly committed to prison every year (2).

Improving prison health has become a World Health Organization priority, given the broad implications for both incarcerated populations and community public health (4, 5). Overcrowded prisons increase infectious disease transmission, suicidality and violence between imprisoned individuals (6, 7). Moreover, prison-related health burdens are considered a structural determinant of community health (8), as mass incarceration is associated with worsened physical and mental health outcomes and heightened social vulnerability in nearby populations (8–10).

The United Nations has alerted about critical issues such as overcrowding and limited access to health services across prisons in Latin America (11), where chronic underfunding, corruption, and organized crime also contribute to conditions that foster violence within facilities (12, 13). In the region, external causes of death, such as homicide, suicide or accidents, among imprisoned people are of particular concern. Suicide incidence is nearly four times higher than in the general population (14), and prison riots and homicides have been reported during the COVID-19 pandemic (12, 15). In Chile, roughly one out of three deaths during imprisonment is by homicide (12), and suicide incidence is twice as high as in the general population (16).

People with criminal justice involvement are more likely to die by homicide than community-based populations (17, 18). Research on prison violence focused on predicting deviant behaviors suggests that individuals convicted of sexual offences or homicide may have elevated levels of aggression than others experiencing imprisonment (19, 20). Other individual-level factors associated with violence are nationality or origin, mental disorders, community deprivation, and antisocial personality traits (7, 21). Contextual factors related to behavioral infractions include overcrowding, high levels of criminal activity, and high-security settings (7, 17). On the other hand, suicide and self-harm are also more prevalent among imprisoned people than in the general population (16, 17). Individual risk factors for suicide attempts include suicidal ideation, prior self-harm, and mental disorders, while contextual factors are also overcrowding, solitary confinement, victimization, and poor social support (6, 22). Furthermore, individuals who engage in dual harm (self-harm/interpersonal aggression) are responsible for nearly three-quarters of misconduct incidents in prisons, presenting a high risk for themselves and others (23).

In Chile, evidence is lacking on the incidence of homicide and of the composite category of external or non-natural deaths in prison, a useful category given the interrelatedness of interpersonal violence and self-harm in these settings (23). Moreover, further evidence is needed on conditions potentially associated with external causes of death during imprisonment, particularly across regions, as mortality risks may vary due to the geographical characteristics of the country. This study aimed to describe the incidence, geographic clustering, associated ecological factors, and changes over time of external causes of death in Chilean prisons, during the period 2000 to 2024. For this purpose, the category ‘external causes of death’ (ECD) is used to represent non-natural deaths in prison, including intentional homicide, suicide, and accidents/other, as defined by the United Nations Office on Drugs and Crime.

## Materials and methods

### Study design

The study has an ecological spatio-temporal design, encompassing the 2000–2024 period across the 16 administrative regions of Chile, which were grouped into northern, central, and southern macrozones. **Appendix** (p 2) details the regions included in each macrozone.

Incidence rates of ECD, homicide and suicide among imprisoned people are reported at the national and regional levels. Excess mortality due to ECD in prison was estimated through Incidence Rate Ratios (IRR) with the general population as reference. Temporal changes in ECD were analyzed with autoregressive models. Latent structures underlying ECD were explored using Principal Component Analysis (PCA) and linear regression models. All statistical analyses were conducted using Stata v.19 (24).

### Data sources and extraction

#### Administrative data extraction

Access to ECD data was obtained through requests via the Transparency Law submitted to the Chilean national prison administration, Gendarmería de Chile, between 2024 and 2025. Data were extracted into Excel matrices (Microsoft Corporation, Redmond, WA, USA; version 16.100.1). Extracted variables included the annual number of homicides, suicides, and all-cause mortality among imprisoned people for the period 2000–2024. Direct links to the data sources and provider organizations for all datasets used are presented in the **Appendix** (p 3).

#### Public data extraction

Public data from Gendarmería de Chile were used to extract the number of incarcerated individuals under closed-regime supervision from 2000 to 2024. Homicides in the general population were retrieved from the online database of the Centro de Estudios y Análisis de Delito (CEAD) of Chile. Age-standardized suicide incidence rates in the general population were extracted from the World Health Organization database. All-cause mortality data for the general population were obtained from the Vital Statistics Yearbooks of the Instituto Nacional de Estadísticas de Chile (INE). Annual national population estimates were extracted from the United Nations Population Division.

Regionally aggregated variables were obtained from the Compendios Estadísticos of Gendarmería de Chile, including prison occupancy, the proportion of individuals convicted of homicide, the proportion convicted of sexual offences, and the proportion of non-national imprisoned individuals. These variables were inferred based on the last data point (2024).

### Statistical analysis

#### Incidence reporting

To calculate the incidence rates of deaths from homicide, suicide, and all causes, the total number of deaths for each cause across all available years was summed in the numerator, and the person-years of imprisonment for the corresponding years were also summed in the denominator based on the annually reported and the person-years of imprisonment for the corresponding years were summed in the denominator, based on the annually reported average daily full-time prison population. The resulting rate was multiplied by 100,000 to estimate the incidence rate per 100,000 person-years of imprisonment. The same calculations were conducted for the general population.

#### Relative risk

National IRRs for homicides, suicides, and all-cause mortality were estimated by dividing the national incidence rate among imprisoned people by the corresponding national incidence rate in the general population, according to the years reported in each database. Data retrieved for the general population informed homicides and all-cause deaths for the entire population, whereas suicides were age standardized. Reference periods of IRR were Homicide: 2003-2023; Suicide: 2000-2021; All-cause mortality: 2000-2023. National IRR estimates were not adjusted for sex or age because individual-level data were not available. Datasets obtained from national registries only provide aggregated annual counts separated by sex. To account for sex-specific differences, sex-stratified IRRs were estimated. Additionally, given that suicide incidence varies across Chilean regions, with a higher incidence observed in the southern macrozone (25), a sensitivity analysis was conducted by fitting a Poisson regression model adjusted for regional suicide variation to estimate a region-adjusted suicide IRR.

#### Temporal changes

Visual inspection was used to explore the temporal distributions of homicide and suicide incidence among imprisoned people (**Appendix**, p 4). First, national-level charts were generated to identify potential temporal variations in homicide or suicide incidences. Following this, regional charts were explored. When chronological patterns were observed across regions, combined charts with overlapping time-slopes were created to compare potential trends.

First-order Prais–Winsten autoregressive models were conducted at the national and regional levels to analyze temporal changes in the incidence of homicides and suicides among the imprisoned people and to estimate potential time effects. In time series data, residuals are often autocorrelated, breaching the ordinary least squares regression assumption of independent errors. The Prais-Winsten method estimates the autocorrelation with the parameter *ρ*, ‘rho’, and uses the estimate to transform the outcome and predictor variables, removing its effect when a linear regression model is fitted (26). This approach enables valid inference on temporal changes and effects while adjusting for first-order autocorrelation.

#### Exploration of latent structures in external causes of death

The administrative regions of Chile were defined as the unit of analysis. A PCA was conducted to reduce dimensionality, given the small sample size (n=16 administrative regions). In this analyses, dependent variables were the incidence of ECD, homicide and suicide (all continuous). Independent variables were contextual factors associated with ECD during imprisonment identified in the literature: regional prison occupancy level (percentage), regional proportion of imprisoned people convicted of homicide, of sexual offences, and proportion of non-national people imprisoned (all continuous). Values for the independent variables were retrieved for the most recent data point (2024). Each independent variable was entered into the PCA to identify potential latent structures. Components with eigenvalues >1.0 were retained, and correlations of the variables with each component were examined. Components were defined based on high loadings and the interpretability of associations, assigning labels accordingly.

Associations between retained components and the incidence of ECD among imprisoned people were tested through linear regression analyses, exploring possible ecological effects. Model selection was based on the adjusted R²-values.

## Results

A database on homicides, suicides, and all-cause deaths among individuals committed to full-time imprisonment in the national correctional system was obtained for the period 2000-2024. A separate database covering deaths from accidents and “other causes” for the period 2015–2024 was also obtained. Data disaggregated by region and sex (men and women) were available only for homicides, suicides, and all-cause deaths from 2000-2023; therefore, deaths from accidents/others are reported solely at the national level for the overall imprisoned population. Sex-specific national incidence rates are provided in the **Appendix** (p 5).

To maintain completeness of the reporting periods and account for regional differences, subsequent ECD estimates and analyses include only homicides and suicides in prisons.

### Incidence of external causes of death among imprisoned people

Between 2000 and 2024, the national registries of Chile reported 974 homicides, 469 suicides, and 3,217 deaths of any cause among imprisoned people. Over this period, across 1,059,116 person-years of imprisonment, the homicide incidence rate was 92 (95% CI: 86-98), the suicide incidence rate was 44 (95% CI: 40-48), the ECD incidence rate was 136 (95% CI: 129-143), and the all-cause mortality incidence rate was 304 (95% CI: 293-314), all per 100,000 person-years of imprisonment. ECD accounted for 45% of national total mortality in the imprisoned population. Mortality data separated by sex were available only for 2000-2023. During this period, women accounted for 0.4% (n=4) of all homicides (n=926); 5% (n=23) of all suicides (n=439), and 3% (n=99) of all deaths (n=3 024). Between 2015 and 2024, 17 prison accidents resulted in death, while 85 deaths were classified as “other causes” (**Appendix**, p 5).

Incidence rates of ECD by region were estimated based on the years for which data on the imprisoned population were available in each region (**Table 1**). Between 2000 and 2023, incidence rates of ECD were 86 (95% CI: 74-99) in northern, followed by 142 (95% CI: 133-151) in central, and 148 (95% CI: 127-170) in southern regions. Homicide incidence rates were 57 (95% CI: 47-67) in northern, 104 (95% CI: 96-111) in central, and 73 (95% CI: 58-88) in southern regions. Suicide incidence rates were 29 (95% CI: 22-36) in northern, 38 (95% CI: 34-43) in central, and 75 (95% CI: 60-90) in southern regions. All estimates are reported per 100,000 person-years of imprisonment. ECD accounted for at least 25% of total mortality, with 65% as the highest proportion of ECD in the Los Ríos region, part of the southern macrozone. Regional incidence rates of all-cause mortality are reported in the **Appendix** (p 6).

**Table 1:** Characteristics of prison settings and incidence rates of external causes of death among imprisoned people, by region^a^.

Macrozone	Region	Regional ecological characteristics of prison settings				Regional incidence rates of external causes of death^b^									
		Prisoners convicted of homicide (%)	Prisoners convicted of sexual crimes (%)	Prison occupancy	Non-national prisoners (%)	Person-years^c^	Number of homicides	Homicide incidence rate	Number of suicides	Suicide incidence rate	Number of external causes of death^d^	External causes of death incidence rate	Relative importance (ref. All-cause mortality)		Incidence rates of external causes of death across regions
													Hom (%)	Suic (%)	
North	Arica y Parinacota	8	6	114	33	32,213	18	56	8	25	26	81	23	10	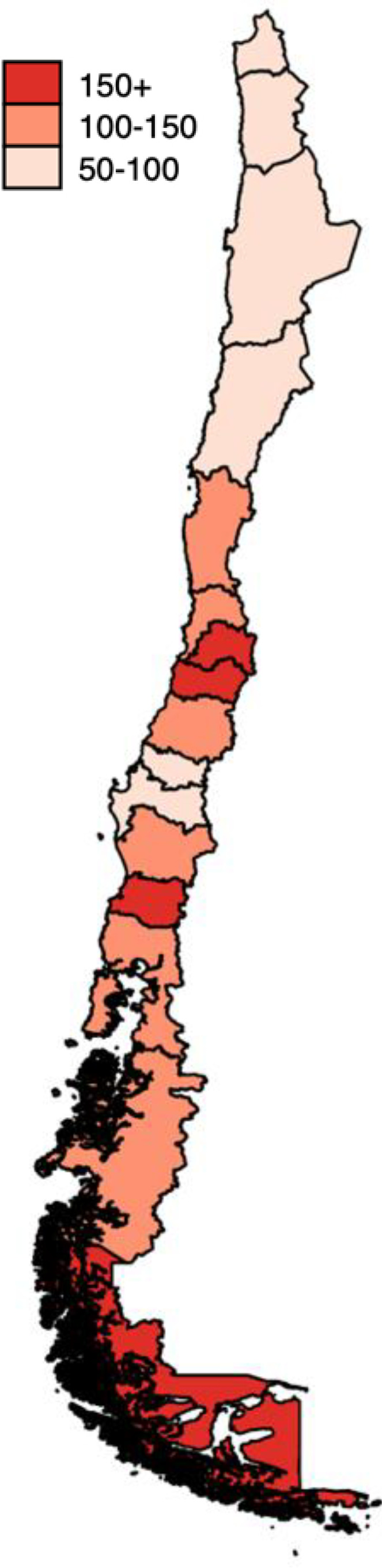
	Tarapacá	6	7	89	50	67,533	23	34	11	16	34	50	20	9	
	Antofagasta	9	7	154	40	48,809	34	70	12	25	46	94	29	10	
	Atacama	7	8	238	18	20,231	6	30	6	30	12	59	19	19	
	Coquimbo	15	7	110	11	44,772	41	92	25	56	66	147	35	21	
Central	Valparaíso	9	9	186	8	107,713	100	93	41	38	141	131	31	13	
	RM	12	6	156	16	379,211	487	128	119	31	606	160	36	9	
	Lib. B. O’ Higgins	13	9	111	9	54,276	40	74	47	87	87	160	26	31	
	Del Maule	11	9	220	5	51,123	30	69	23	45	53	104	23	19	
	Ñuble	9	12	167	9	4,790	0	NA	3	63	3	63	0	25	
	Biobío	15	8	105	5	86,415	51	59	30	35	81	94	28	17	
South	La Araucanía	12	17	167	3	45,563	31	68	19	42	50	110	31	19	
	Los Ríos	18	14	90	5	18,691	31	166	16	86	47	251	43	22	
	Los Lagos	16	16	109	7	48,308	28	58	40	83	68	141	22	32	
	Aysén	18	26	120	10	5,860	2	34	4	68	6	102	20	40	
	Magallanes y Antártica	15	22	110	14	8,301	1	12	16	193	17	205	3	44	

Source: Authors’ own calculations based on data from Gendarmería de Chile (**Appendix**, p 3). RM, Región Metropolitana; NA, Not Applicable. ^a^Regional data are reported for the period 2000-2023, except for the Los Ríos, Arica y Parinacota (2008–2023) and Ñuble (2018–2023) regions. ^b^Death incidences are multiplied per 100,000 person-years of imprisonment. ^c^Person-years refer to the total sum of the annual average number of imprisoned people for each year considered. ^d^ECD were calculated by summing the number of homicides and suicides. Chile, 2000—2023.

### Relative risk of external causes of death among imprisoned people

National IRRs were estimated by comparing the imprisoned population with the general population (**Table 2**). The relative risk of homicide among imprisoned individuals was higher for males (IRR: 16; 95% CI: 15-17). Imprisoned people also had a fourfold higher risk of suicide than the general population (IRR: 4; 95% CI: 4-5). Imprisoned women had a substantially higher relative risk of suicide (IRR: 9; 95% CI: 5-14). The incidence of all-cause mortality was lower among imprisoned females (IRR: 0.25; 95% CI: 0.20-0.30) and males (IRR: 0.52; 95% CI: 0.50-0.54) compared to the general population. Total IRRs should be interpreted with caution due to differences in the sex distribution between imprisoned people and the general population. A sensitivity analysis estimating the national suicide IRR, adjusted for region-specific suicide incidence to account for Chile’s geographical variation, showed no substantial differences compared with the main analysis (**Appendix**, p 7).

**Table 2:** National Incidence Rate Ratios (IRR) for external causes and all-cause deaths^a^.

Sex	Homicide	Suicide	All-cause mortality
	IRR (95% CI)	IRR (95% CI)	IRR (95% CI)
Men	16 (15 – 17)	2 (2 – 2)	0.52 (0.50 – 0.54)
Women	6 (0.14 – 31)	9 (5 – 14)	0.25 (0.20 – 0.30)
Total	29 (27 – 31)	4 (4 – 5)	0.53 (0.51 – 0.55)

Source: Authors’ own estimations based on data from Gendarmería de Chile and publicly available databases (**Appendix**, p 3). IRR, Incidence Rate Ratios; 95% CI, 95% Confidence Intervals. ^a^Complete information and the periods considered to estimate IRRs are presented in the **Appendix** (pp 8-9).

Comparison between imprisoned people and general population. Chile.

### Changes over time in external causes of death from 2000 to 2024

**Figure 1:**
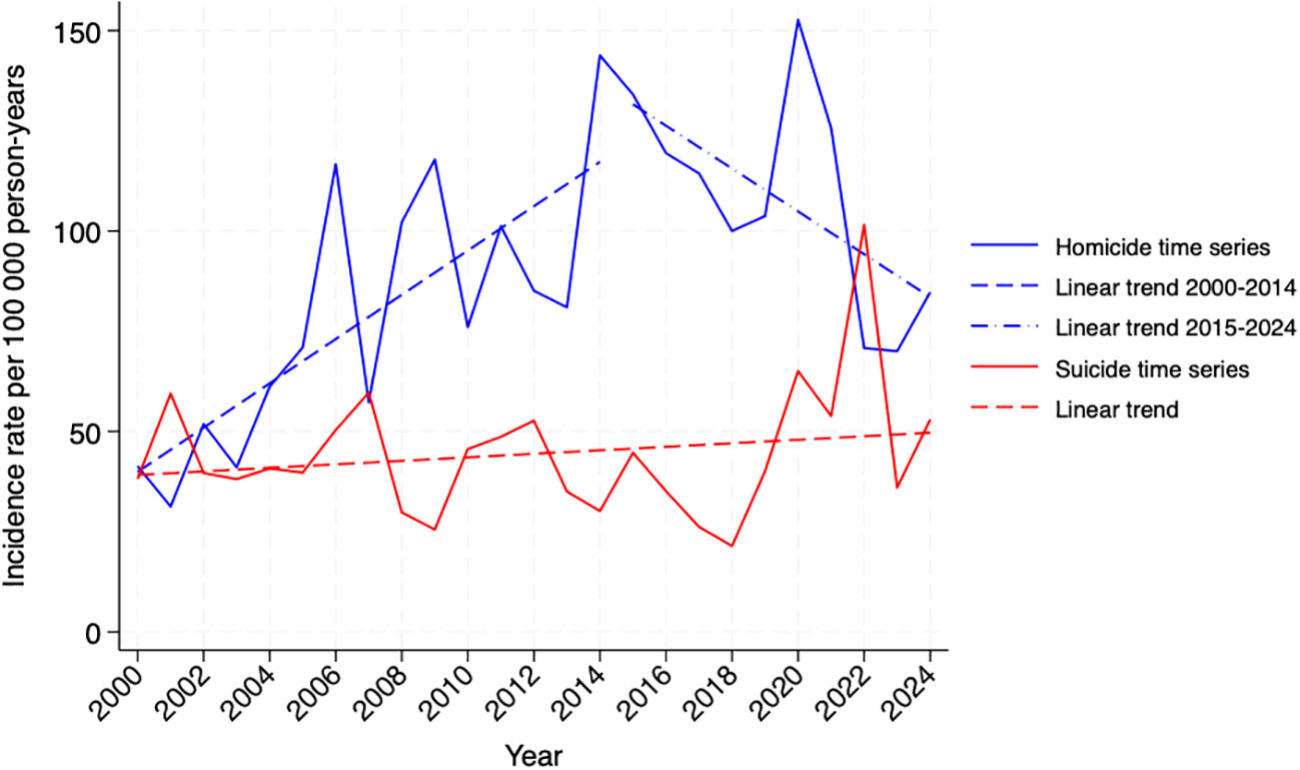
Time series of homicide and suicide incidence rates among imprisoned people. Chile, 2000-2024. Source: Authors’ own estimations based on data from Gendarmería de Chile (**Appendix**, p 3).

**Figure 1** presents national-level changes over time in the incidence of ECD among imprisoned people. The homicide series exhibits abrupt fluctuations, indicating a possible upward slope. The suicide series shows random oscillations over the years, suggesting a stable slope.

The results of the temporal analysis using first-order Prais–Winsten models, conducted at both national and regional levels, are reported in the **Appendix** (pp 10-11). An initial model reveals an upward slope in the homicide incidence rate during the period 2000-2014 (ß=5.36, p<0.0001). A second model was fitted for the full period (2000–2024), incorporating a time-period interaction term to identify a potential trend break, which revealed a reversal in the slope from 2015 onward. This model was also adjusted for the pandemic effect of years 2020 and 2021, which appeared to exhibit a transitory peak in rates. After the 2015 trend break, the homicide incidence slope shifted from an annual increase of 5.4 points (ß=5.43, p<0.0001) to a yearly decline of -6.8 points (ß=–12.26, p<0.0001). The national suicide incidence rate showed no significant change over the 2000–2024 period (ß=0.44, p=0.385).

**Figure 2:**
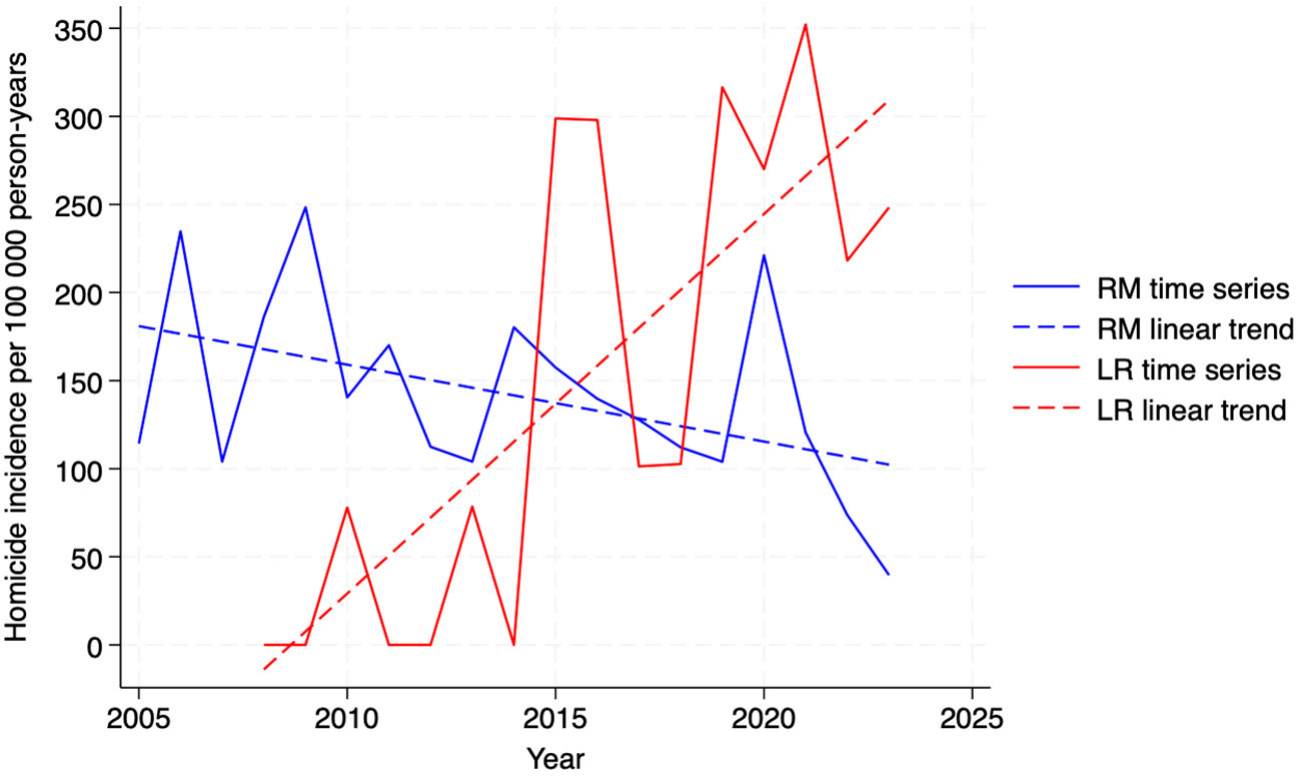
Time series of homicide incidence rates among imprisoned people in the Región Metropolitana (RM) and Los Ríos (LR). Chile, 2005-2024. Source: Authors’ own estimations based on data from Gendarmería de Chile (**Appendix**, p 3).

The regional analysis shows that since 2005, the homicide incidence rate in the Region Metropolitana has followed a decreasing slope, adjusting for the effect of the COVID-19 pandemic in 2020 (ß=–5.80, p=0.001). Statistically significant increases in homicide incidence over time were observed in the regions of Tarapacá (ß=4.10, p=0.005), Coquimbo (ß=4.10, p=0.029), Valparaíso (ß=5.86, p=0.012), O’Higgins (ß=7.46, p=0.006), La Araucanía (ß=5.31, p=0.008), Los Ríos (ß=21.57, p=0.000), and Los Lagos (ß=4.84, p=0.028). Remaining regions showed no change over time (**Appendix**, pp 4, 10-11). The most pronounced increase occurred in the Los Ríos region, which maintained a strong upward slope even after adjusting for the pandemic effect (ß=19.28, p=0.002). **Figure 2** presents the intersection of slopes between the Metropolitan and Los Ríos regions. The suicide incidence across regions did not change significantly throughout the 2000–2023 period (**Appendix**, pp 10-11).

### Exploratory analysis of latent structures in external causes of death

The PCA identified two potential patterns to explain the incidence of external causes of death in prisons. Component 1 was characterized by high loadings for the variable regional proportion of individuals convicted of homicide (positive correlation), convicted of sexual offences (positive correlation), and the proportion of non-national individuals in prison (negative correlation). This component was labelled “Imprisoned nationals convicted of homicide or sexual offences”. Component 2 showed high loadings for regional prison occupancy (positive correlation) and the proportion of non-national imprisoned people (negative correlation), being labelled “Imprisoned nationals in overcrowded settings”. These components explained 55% and 29% of the variance, respectively, accounting for 84% of the total variance in the identified structural patterns. Eigenvalues and loadings for each component are presented in the **Appendix** (p 12).

**Figure 3:**
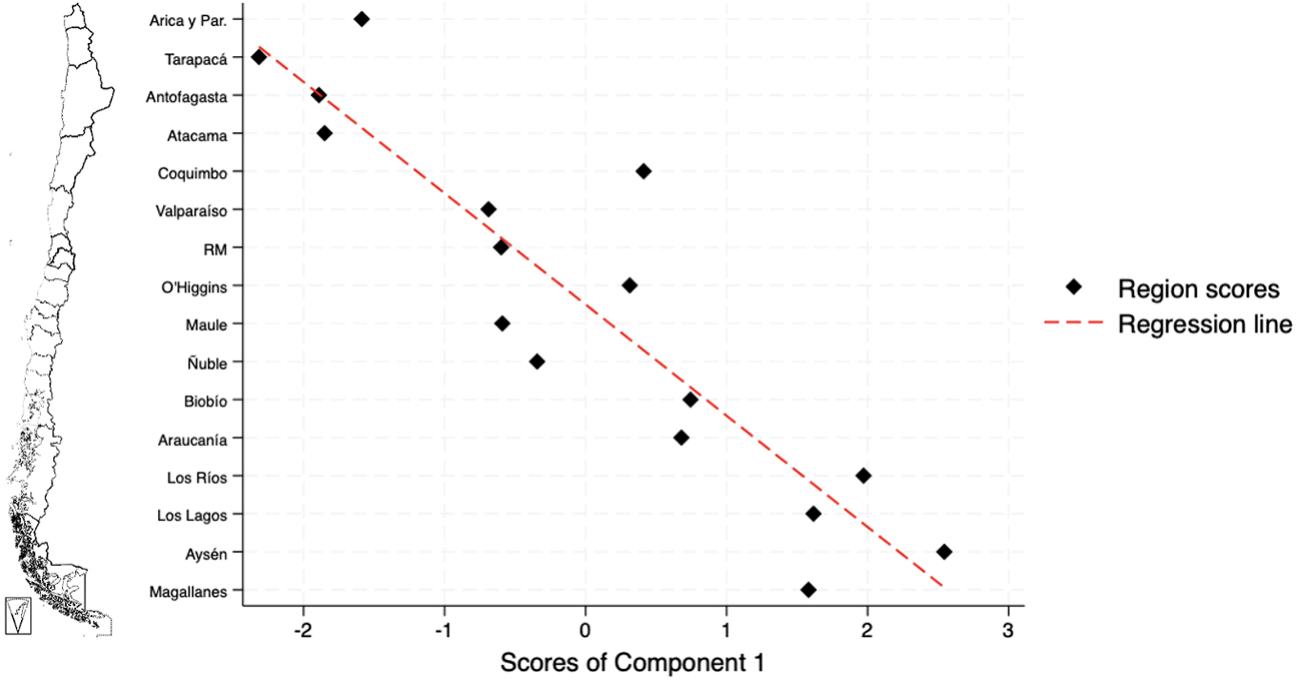
Regional distribution of scores for Component 1: Imprisoned nationals convicted of homicide or sexual offences; regions are presented in north-to-south geographical order.

The components were included in linear regression models to explore their effects on the regional incidence of homicide and suicide among imprisoned people (**Table 3**). Univariable models explained a larger proportion of the observed variability, according to adjusted R² values (**Appendix**, p 12). The analyses showed a statistically significant increase in external causes of death (ß=23.136, p=0.009) and suicide incidence rates in regions with higher scores for Component 1 (ß=18.63, p=0.007). No association was found between Component 2 and mortality outcomes. Homicide incidence was not significantly associated with either component in this analysis. The distribution of Component 1 scores across regions was explored, revealing higher scores in the southern regions of the country (**Figure 3**).

**Table 3:** Linear regression analyses of homicide and suicide incidence among imprisoned people using principal components. Chile, 2000-2023^a^ .

Independent variables (components)	Homicide incidence rate			Suicide incidence rate			External causes of death incidence rate		
	Coefficient (ß)	95% CI	P-value	Coefficient (ß)	95% CI	P-value	Coefficient (ß)	95% CI	P-value
Univariable models									
Comp. 1. Imprisoned nationals convicted of homicide or sexual offences	4.497	-11.454 to 20.449	0.555	18.638	6.052 to 31.224	0.007	23.136	6.819 to 39.452	0.009
Comp. 2. Imprisoned nationals in overcrowded settings	-2.809	-24.855 to 19.236	0.789	-2.932	-25.453 to 19.588	0.784	-5.741	-34.316 to 22.833	0.673
Multivariable model^b^									
Comp. 1. Imprisoned nationals convicted of homicide or sexual offences	4.497	-12.131 to 21.126	0.569	18.638	5.545 to 31.731	0.009	23.136	6.266 to 40.005	0.011
Comp. 2. Imprisoned nationals in overcrowded settings	-2.809	-25.556 to 19.938	0.794	-2.932	-20.843 to 14.978	0.729	-5.741	-28.818 to 17.334	0.600

Source: Authors’ own estimations based on data from Gendarmería de Chile (**Appendix**, p 3). 95% CI, 95% Confidence Intervals. ^a^Regional data used in the linear regression analyses included the period 2000-2023, except for the Los Ríos, Arica y Parinacota (2008-2023) and Ñuble (2018-2023) regions. ^b^In multivariable models, Component 1 was the independent variable adjusted for Component 2.

This distribution reflects higher proportions of imprisoned nationals and individuals convicted of homicide or sexual offences in the southern macrozone, a pattern associated with elevated incidence of ECD within prison settings. The northern regions of Tarapacá and Antofagasta exhibit the lowest Component 1 scores, whereas the regions of Aysén and Los Ríos the highest. Region-specific scores of Component 1 are presented in the **Appendix** (p 13).

## Discussion

In Chile, the incidence rates of homicide and suicide among imprisoned people were 92 and 44 cases per 100,000 person-years of imprisonment, respectively. Nationally, ECD accounts for 45% of all prison deaths. Temporal variations suggest an increase in homicide incidence in specific regions. There is a higher concentration of suicides in prisons located in the southern regions of the country.

ECD in prison constitute a major public health challenge. Because ECD are potentially preventable, an important proportion of mortality in Chilean prisons may be accessible through prevention measures. Imprisoned men in Chile present a risk of homicide up to 16 times higher than that of men in the general population, even though government authorities hold control over and responsibility for the safety of the prison environment. In some southern regions of the country, ECD account for over 60% of total mortality. Low rates of deaths from other causes may be explained by the practice of changing terminally ill individuals to alternative sentences. The high incidence of ECD in prison is mainly attributable to risk factors imported into prison, since higher ECD rates were documented in this population while living in the community (5, 6, 18). Imprisonment could constitute a window of opportunity to intervene in risk pathways leading to elevated mortality. There are established interventions for suicide (17, 27, 28) and interpersonal violence prevention during incarceration (29, 30). Strategies targeting contextual factors of prison violence include reducing overcrowding, improving infrastructure, decentralizing people with high-security needs, and diminishing the influence of organized crime (7, 17). Reducing violence within facilities, fomenting social support, and limiting solitary confinement are key to lowering suicide rates (17, 22). Prison staff play a crucial role in ECD prevention. Optimal relationships between imprisoned people and staff create an environment of trust that prevents violence (12). Given that violence perpetration also occurs by staff, increased oversight and better training in peaceful conflict resolution are required (12). Additionally, suicide risk assessment can be improved (31).

Homicides among imprisoned people showed temporal changes across regions, while suicide incidence remained stable. At the national level, the COVID-19 pandemic marked a transitory break in the declining slope of homicides, with a marked increase between 2020 and 2021, possibly explained by riots and collective fights that occurred during this period (15). An intermittent increase in suicides potentially associated with the COVID-19 pandemic was also observed, a phenomenon previously reported in other prison settings which was possibly linked to heightened psychological distress and the emerging restrictions on receiving visits from relatives (32). Temporal changes of homicide incidence across regions reflect both the escalation of prison violence in specific settings and the policies adopted to mitigate it. A possible explanation for the rise in homicides in southern regions may relate to the emergence of new gangs; it has been suggested that violence escalates during their imposition and stabilizes once consolidation is reached (12). The study findings on regional differences in the evolution of homicides also support previous reports that prison transfers can increase violence (12). In Chile, disruptive individuals have been transferred to distant southern units as a punitive measure for misconduct (33). Relocations may explain the decline in homicide incidence in the Metropolitan Region since 2015 and the simultaneous increase in the Los Ríos region. This outcome is chronologically related to a reported increase in transfers of individuals with disruptive conduct across regions in 2017 (12). Although limiting the concentration of people with high security needs has been recommended (7), poor implementation of this policy may increase violence in the receiving facilities, reinforcing the need to review distribution mechanisms (12).

Prevention efforts must also incorporate a gender approach, since structural differences need to be considered (31). Imprisoned women face a higher relative risk of suicide than men when compared to the general population, while nearly all homicides occur among the male prison population; both patterns are consistently reported in the literature (16, 34). In Chile, prison regulations are applied uniformly across the incarcerated population, regardless of sex (3). 70% of imprisoned women have underage children, and many of them care for their infants in sections for nursing children, while others serve sentences during pregnancy (3).

Regions with a higher concentration of imprisoned nationals and individuals convicted of homicide or sexual crimes exhibit higher incidence of ECD, an effect possibly explained by the geographical distribution of suicide incidence. This finding raises new questions about the factors underlying this association. A plausible contextual hypothesis is that the high concentration of individuals convicted of violent crimes creates a hostile prison environment, increasing interpersonal violence and thereby raising the risk of both suicide and homicide. Individuals who commit homicide while incarcerated often have a prior history of homicide and behavioral maladjustment during imprisonment (20). Furthermore, perpetrators of sexual offenses are often victims of violent aggression during imprisonment due to the unacceptability of those crimes among criminal subcultures dominating life in prison (19). Violence has been more prevalent in facilities with higher proportions of individuals with high-security needs (7), a pattern also observed in Chilean prisons (35). Hostile prison environments may trigger suicide risk, such as low levels of social support and high levels of victimization (22). This contextual hypothesis suggests a shared pathway of violence that underlies both homicide and suicide mortality in prison.

Nationality may also be associated with external causes of death in prisons (21); informal reports from Chile mention that non-national gang members entering prisons can exacerbate violence (12). However, the findings related to Component 1 “Imprisoned nationals convicted of homicide or sexual offences” geographical distribution, suggest that higher proportions of non-national people in prisons are not correlated with increased homicide or suicide incidence. This indicates that violence can originate from specific non-national individuals with a history of criminal records rather than the immigrant population as a whole.

The sensitivity analysis conducted provides evidence to exclude the possibility that the national suicide IRR is inflated by the higher suicide incidence in the general population of the southern macrozone. However, in this analysis, regional suicide incidence in general population was used as a proxy to control for territorial differences, capturing only part of the influence of external factors. Other variables, such as meteorological conditions or access to mental health care may still affect the elevated suicide incidence observed in prisons in southern regions. For example, meteorological factors can act as confounders in the association between other variables (e.g., mental health) and suicide (36), potentially explaining the higher suicide incidence observed in southern prisons with higher latitude, where exposure to sunlight is lower (37). Future research should further investigate the influence of these structural and environmental factors to better understand the excess of suicide incidence among imprisoned populations compared with the community.

## Limitations

ECD are difficult to categorize; suicides may be misclassified as accidents (16), and homicides are often underreported (17). Some individuals may be misclassified as unknown, others, or awaiting classification for the time of investigation. This study analyzed aggregated variables, and thus inferences should be made at the level of regional clusters rather than individual-level risk factors. The small number of observations (n=16 regions) limits the interpretability of the PCA; therefore, a small number of variables was included to avoid overfitting. This limitation and the interpretation of ecological variables may explain the absence of other significant associations in the linear regression analyses, such as the well-established relationship between overcrowding and increased homicide rates (7, 17). The IRRs compared populations with different age structures, and should be interpreted with caution, although it is unlikely that the large differences in incidence are only explained by age composition. Given that the size of the imprisoned female population is typically small (2), the number of mortality events was low. Therefore, the resulting incidence and IRR estimates for women were unstable, with wide confidence intervals, and should be interpreted with caution.

## Conclusions

This study showed high incidence rates of ECD within the correctional system of Chile, highlighting the importance of framing prison mental health and violence as major public health concerns. A downward trend in homicide incidence since 2015 from high levels is a positive start. However, the disproportionate burden of ECD among imprisoned people calls for the need to develop more effective strategies. Focused prevention policies for suicide are needed in regions with a high concentration of individuals convicted of violent offences and for homicide. Interventions should incorporate evidence-based practices to prevent violent and suicidal behavior in prisons, including screening, staff training in monitoring, supervision and de-escalation, mental health interventions, and postvention measures (38). A scientific research agenda on prison violence and mortality in Latin America should be promoted, as other countries in the region may exhibit even higher homicide rates than Chile (12, 15, 39). Future studies should investigate additional ecological and individual-level risk factors associated with ECD across countries and regions (17). Improving data transparency, through regular publication of updated mortality incidence rates, is recommended to allow the evaluation of the effectiveness of implemented interventions.

## Data Availability

The data analyzed in this study is subject to the following licenses/restrictions: Institutional restrictions. However, interested researchers can obtain access via Transparency Council requests from Gendarmería de Chile, the national prison administration of Chile. Requests to access these datasets should be directed to https://www.gendarmeria.gob.cl/tramite13.html.

## References

[1] World Prison Brief (2025). Available online at: https://www.prisonstudies.org/country/Chile (Accessed July 2, 2025).

[2] Gendarmería de Chile . Compendio Estadístico Penitenciario. Santiago, Chile: Gendarmería de Chile (2024).

[3] Instituto Nacional de Derechos Humanos . Informe Anual 2024; Situación de los Derechos Humanos en Chile. Santiago, Chile: Instituto Nacional de Derechos Humanos (2024).

[4] World Health Organization . Prisons and Health. Copenhagen, Denmark: World Health Organization (2014).

[5] Borschmann R Borschmann R Keen C Spittal MJ Preen D Pirkis J . Rates and causes of death after release from incarceration among 1 471 526 people in eight high-income and middle-income countries: an individual participant data meta-analysis. Lancet. (2024) 403:1779–88. doi: 10.1016/S0140-6736(24)00344-1, PMID: 38614112

[6] Brinkley-Rubinstein L Berk J Williams BA . Carceral health care. N Engl J Med. (2025) 392:892–901. doi: 10.1056/NEJMra2212149, PMID: 40009808 PMC11995879

[7] Gonçalves LC Gonçalves RA Martins C Dirkzwager AJE . Predicting infractions and health care utilization in prison:A meta-analysis. Criminal Justice Behavior. (2014) 41:921–42. doi: 10.1177/0093854814524402

[8] Larrabee Sonderlund A Wang EA Williams NJ Horowitz CR Schoenthaler A Holaday LW . County incarceration rate and stroke death: A cross-sectional study of the influence of physical environment, health care access, and community mental distress. J Am Heart Assoc. (2025) 14:e039065. doi: 10.1161/JAHA.124.039065, PMID: 40357666 PMC12184567

[9] Hickson A Purbey R Dean L Gallo JJ Thorpe RJ Pollack Porter K . A consequence of mass incarceration: county-level association between jail incarceration rates and poor mental health days. Health Justice. (2022) 10:31. doi: 10.1186/s40352-022-00194-6, PMID: 36269431 PMC9587611

[10] Holaday LW Howell B Thompson K Cramer L Wang EA . Association of census tract-level incarceration rate and life expectancy in New York State. J Epidemiol Community Health. (2021) 75:1019–22. doi: 10.1136/jech-2020-216077, PMID: 33906904 PMC9052201

[11] United Nations . Immediate action needed to address conditions of detention in Latin America: United Nations, United Nations Human Rights Office of the High Comissioner (2012). Available online at: https://www.ohchr.org/en/stories/2012/02/immediate-action-needed-address-conditions-detention-latin-america (Accessed July 2, 2025).

[12] Sanhueza GE Pérez F Candia J Urquieta MA . Inmate-on-inmate prison violence in Chile: the importance of the institutional context and proper supervision. J Interpersonal Violence. (2021) 36:NP13391–NP414. doi: 10.1177/0886260520906177, PMID: 32081065

[13] Dammert L . Rehabilitation in Latin America: Could innovation be fostered in precarious conditions? (2016), 1–13.

[14] Fritz FD Fazel S Benavides Salcedo A Henry P Rivera Arroyo G Torales J . 1324 prison suicides in 10 countries in South America: incidence, relative risks, and ecological factors. Soc Psychiatry Psychiatr Epidemiol. (2021) 56:315–23. doi: 10.1007/s00127-020-01871-3, PMID: 32405788 PMC7618062

[15] de Oliveira Andrade R . Covid-19: Prisons exposed in Brazil’s crisis. Bmj. (2020) 370:m2884. doi: 10.1136/bmj.m2884, PMID: 32694123

[16] Mundt AP Cifuentes-Gramajo PA Baranyi G Fazel S . Worldwide incidence of suicides in prison: a systematic review with meta-regression analyses. Lancet Psychiatry. (2024) 11:536–44. doi: 10.1016/S2215-0366(24)00134-2, PMID: 38823401

[17] Fazel S Hayes AJ Bartellas K Clerici M Trestman R . Mental health of prisoners: prevalence, adverse outcomes, and interventions. Lancet Psychiatry. (2016) 3:871–81. doi: 10.1016/S2215-0366(16)30142-0, PMID: 27426440 PMC5008459

[18] Willoughby M Young JT Spittal MJ Borschmann R Janca E Kinner PSA . Violence-related deaths among people released from incarceration: systematic review and meta-analysis of cohort studies. EClinicalMedicine. (2021) 41:101162. doi: 10.1016/j.eclinm.2021.101162, PMID: 34746721 PMC8551597

[19] van den Berg C Beijersbergen K Nieuwbeerta P Dirkzwager A . Sex offenders in prison: are they socially isolated? Sexual Abuse. (2018) 30:828–45. doi: 10.1177/1079063217700884, PMID: 28372519

[20] Reidy TJ Sorensen JR Bonner HS . Prison homicide: an extension of violent criminal careers? J Interpers Violence. (2020) 35:5676–90. doi: 10.1177/0886260517721895, PMID: 29294859

[21] Butler M McNamee CB Kelly D . Risk factors for interpersonal violence in prison: evidence from longitudinal administrative prison data in northern Ireland. J Interpers Violence. (2022) 37:Np14610–np32. doi: 10.1177/08862605211006363, PMID: 33847147 PMC9326804

[22] Favril L Shaw J Fazel S . Prevalence and risk factors for suicide attempts in prison. Clin Psychol Rev. (2022) 97:102190. doi: 10.1016/j.cpr.2022.102190, PMID: 36029609

[23] Shafti M Pratt D Taylor P Forrester A . The duality of self-harm and aggression: implications for research and practice. BJPsych Bull. (2025) 49:219–22. doi: 10.1192/bjb.2025.10088, PMID: 40740041 PMC12314404

[24] StataCorp . Stata Statistical Software: Release 19. College Station, TX: StataCorp LLC (2025).

[25] Departamento de Estadísticas e Información de Salud (DEIS) . Mortalidad por Suicidio por región y grupos de edad, Chile 2000–2017. Santiago, Chile: Departamento de Estadísticas e Información de Salud (DEIS) Ministerio de Salud, Gobierno de Chile (2025). Available online at: https://deis.minsal.cl/ (Accessed December 4, 2025).

[26] Bottomley C Ooko M Gasparrini A Keogh R . In praise of Prais-Winsten: An evaluation of methods used to account for autocorrelation in interrupted time series. Stat Med. (2023) 42:1277–88. doi: 10.1002/sim.9669, PMID: 36722328 PMC10946734

[27] Carter A Butler A Willoughby M Janca E Kinner SA Southalan L . Interventions to reduce suicidal thoughts and behaviours among people in contact with the criminal justice system: A global systematic review. EClinicalMedicine. (2022) 44:101266. doi: 10.1016/j.eclinm.2021.101266, PMID: 35072018 PMC8763634

[28] Stijelja S Mishara BL . Preventing suicidal and self-Injurious behavior in correctional facilities: A systematic literature review and meta-analysis. EClinicalMedicine. (2022) 51:101560. doi: 10.1016/j.eclinm.2022.101560, PMID: 35898320 PMC9309412

[29] Day A Newton D Cooke D Tamatea A . Interventions to prevent prison violence: A scoping review of the available research evidence. Prison J. (2022) 102:745–69. doi: 10.1177/00328855221136201

[30] Day A Newton D Tamatea A . A scoping review of family focussed interventions to prevent prison violence. Int J Offender Ther Comp Criminol. (2023) 67:151–63. doi: 10.1177/0306624X211023917, PMID: 34114483

[31] Slade K Justice L Baguley T Bowen E Shorter GW Adamson L . Mortality after prison release in England and Wales, 2019-2021: A comparative analysis of cause-specific death rates and risk profiles. Soc Sci Med. (2025) 369:117821. doi: 10.1016/j.socscimed.2025.117821, PMID: 39946863

[32] Kola L Kohrt BA Hanlon C Naslund JA Sikander S Balaji M . COVID-19 mental health impact and responses in low-income and middle-income countries: reimagining global mental health. Lancet Psychiatry. (2021) 8:535–50. doi: 10.1016/S2215-0366(21)00025-0, PMID: 33639109 PMC9764935

[33] Instituto Nacional de Derechos Humanos . Situación de los Derechos Humanos en Chile; Informe Anual 2015. Santiago, Chile: Instituto Nacional de Derechos Humanos (2015).

[34] Worthey A Thomas A Jones C Abuzeid A White CQ . Violence in incarcerated populations: a review of the literature. Curr Trauma Rep. (2022) 8:172–8. doi: 10.1007/s40719-022-00234-4

[35] Sanhueza G Miller R . Prison violence in Chilean facilities: a preliminary overview. Rev Española Investigación Criminológica. (2016) 14:1–39.

[36] Cornelius SL Berry T Goodrich AJ Shiner B Riblet NB . The effect of meteorological, pollution, and geographic exposures on death by suicide: A scoping review. Int J Environ Res Public Health. (2021) 18:1–16. doi: 10.3390/ijerph18157809, PMID: 34360101 PMC8345465

[37] An S Lim S Kim HW Kim HS Lee D Son E . Global prevalence of suicide by latitude: A systematic review and meta-analysis. Asian J Psychiatr. (2023) 81:103454. doi: 10.1016/j.ajp.2023.103454, PMID: 36634498 PMC9822839

[38] Cifuentes-Gramajo PA Beigel L Bacigalupo F . Suicide prevention in Latin American prisons: a multiple case study with meta-matrix of policies, programmes and protocols in 17 countries. BMJ Glob Health. (2026) 11(1):e021858. doi: 10.1136/bmjgh-2025-021858, PMID: 41513301 PMC12815102

[39] Mundt AP Rozas-Serri E Asencio Rojas BI Morales-Rojas A Cifuentes-Gramajo PA Alvarado S . Incidence of all-cause mortality in prisons: research protocol for a global registry study and systematic literature review with meta-regression analyses. BMJ Open. (2025) 15(12):e111125. doi: 10.1136/bmjopen-2025-111125, PMID: 41407424 PMC12716586

