# Supplementary material for: Incidence, ecological factors, and temporal trends of prison homicides and suicides in Chile, 2000-2024
Source: Front Psychiatry. 2026 Jan 22;16:1715635. doi: 10.3389/fpsyt.2025.1715635 (PMC12872864; doi:10.3389/fpsyt.2025.1715635)
Supplement: Supplementary file 1 [file DataSheet1.zip › Appendix of Incidence, ecological factors, and temporal trends of prison homicides and suicides in Chile, 2000-2024.DOCX]

**Online Supplement: Incidence, ecological factors, and temporal trends of prison homicides and suicides in Chile, 2000-2024**

Index

[Table 1. Grouping of Chilean administrative regions into macrozones 2](#_Toc215923561)

[Table 2. Data sources and provider organizations 3](#_Toc215923562)

[Figure 1. Temporal changes in homicide incidence among imprisoned people by region 4](#_Toc215923563)

[Table 3. National incidence rates of homicide and suicide among imprisoned people by sex, 2000-2023 5](#_Toc215923564)

[Table 4. National number of deaths by accident and by “other causes” among imprisoned people, 2015-2024 5](#_Toc215923565)

[Table 5. Regional incidence rates of all-cause deaths among imprisoned people 6](#_Toc215923566)

[Table 6. Sensitivity analysis of national suicide Incidence Rate Ratios (IRR) between imprisoned people and the general population: univariable and multivariable region-adjusted Poisson regression models 7](#_Toc215923567)

[Table 7. National Incidence Rate Ratios between imprisoned people and the general population: A) Both sexes; B) Men; C) Women 8](#_Toc215923568)

[Table 8. National and regional changes over time in external causes of death (homicide, suicide) among imprisoned people: Crude first-order Prais-Winstein regression outputs 10](#_Toc215923569)

[Table 9. National and regional trends in homicide among imprisoned people: First-order Prais-Winstein regression outputs adjusted for trend break and the COVID-19 pandemic effects 11](#_Toc215923570)

[Table 10. Principal Component Analysis (PCA) eigenvalues 12](#_Toc215923571)

[Table 11. PCA factor loadings 12](#_Toc215923572)

[Table 12. Linear regression R-squared (R²) for external causes of death (homicide, suicide) and suicide incidence with PCA Component 1 “Imprisoned nationals convicted of homicide or sexual offences” as the independent variable 12](#_Toc215923573)

[Table 13. Component 1: “Imprisoned nationals convicted of homicide or sexual offences” scores by region 13](#_Toc215923574)

# Table 1. Grouping of Chilean administrative regions into macrozones

| **Macrozone** | **Administrative regions** |
| --- | --- |
| **North** | Arica y Parinacota |
|  | Tarapacá |
|  | Antofagasta |
|  | Atacama |
|  | Coquimbo |
| **Central** | Valparaíso |
|  | Region Metropolitana |
|  | Libertador B. O’ Higgins |
|  | Del Maule |
|  | Ñuble |
|  | Biobío |
| **South** | La Araucanía |
|  | Los Ríos |
|  | Los Lagos |
|  | Aysén |
|  | Magallanes y Antártica |

**Note:** Macrozone grouping according to Decree No. 7 of the Ministry of Science, Technology, Knowledge and Innovation (CTCI).

# Table 2. Data sources and provider organizations

| **Extracted data** | **Organization** | **Link** |
| --- | --- | --- |
| Prison homicide, suicide and all-cause deaths | Gendarmería de Chile | Data provided directly by Transparency. |
| Person-years | Gendarmería de Chile | <https://www.gendarmeria.gob.cl/compendios.html> |
| General population homicides | Centro de Estudios y Análisis de Delito (CEAD), Ministerio del Interior, Chile | <https://cead.spd.gov.cl/estadisticas-delictuales/> |
| General population suicides | World Health Organization (WHO) | <https://www.who.int/data/gho/data/indicators/indicator-details/GHO/age-standardized-suicide-rates-(per-100-000-population> |
| General population all-cause deaths | Instituto Nacional de Estadísticas de Chile (INE) | <https://www.ine.gob.cl/estadisticas/sociales/demografia-y-vitales/nacimientos-matrimonios-y-defunciones> |
| Total population country | United Nations – Population Division | <https://population.un.org/dataportal/home?df=42518f95-7497-4c79-ac8a-94b7a811025f> |
| Prison occupancy by administrative region | Gendarmería de Chile | <https://www.gendarmeria.gob.cl/compendios.html> |
| Proportion of prisoners convicted of homicide by administrative region | Gendarmería de Chile | <https://www.gendarmeria.gob.cl/compendios.html> |
| Proportion of prisoners convicted of sexual offences by administrative region | Gendarmería de Chile | <https://www.gendarmeria.gob.cl/compendios.html> |
| Proportion of non-national prisoners by administrative region | Gendarmería de Chile | <https://www.gendarmeria.gob.cl/compendios.html> |
| General population suicide incidence across regions | Departamento de Estadística e Información de Salud (DEIS) | <https://deis.minsal.cl/> |

#
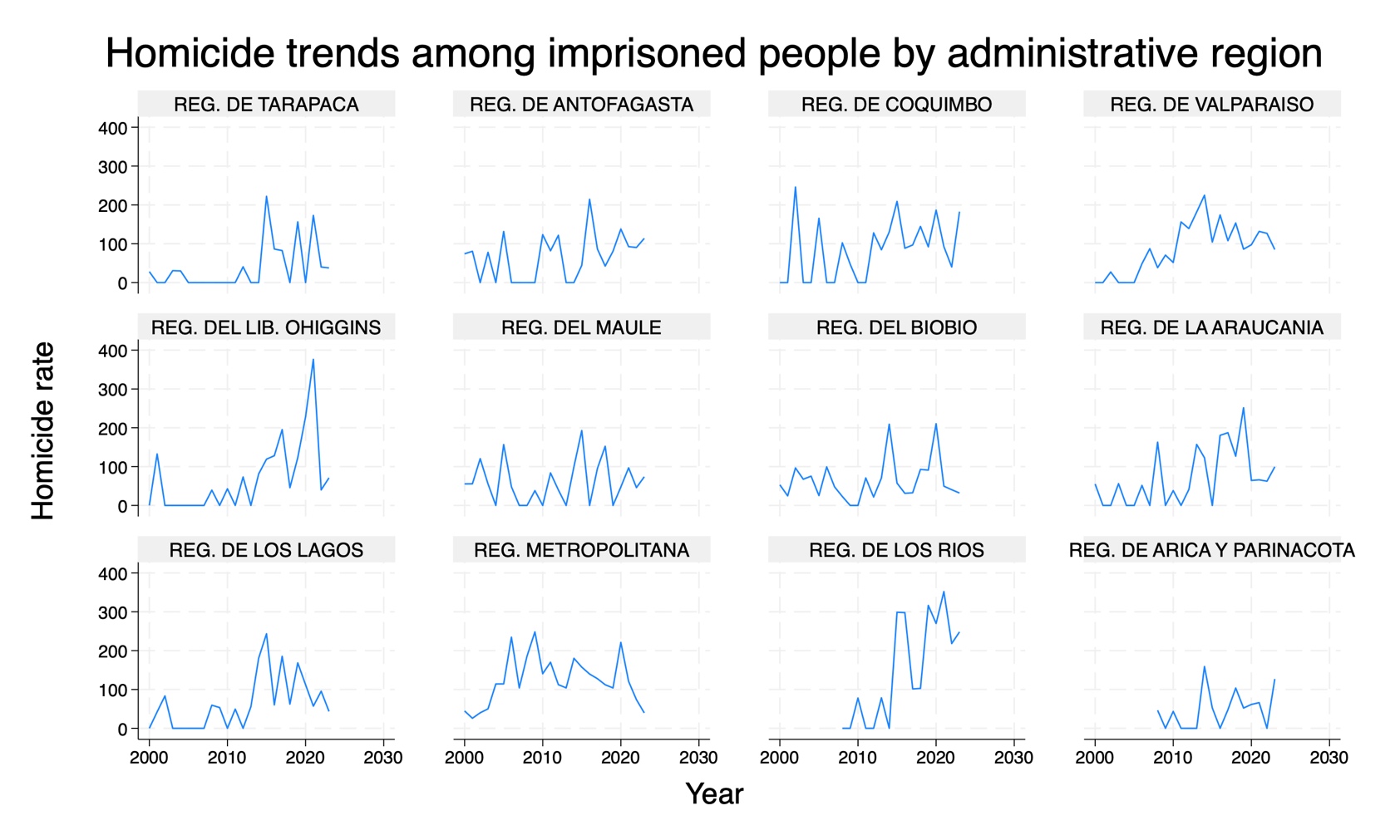
Figure 1. Temporal changes in homicide incidence among imprisoned people by region

Only regions with ≥15 cases were considered in the trend analysis. Suicide trend graphs are not presented as no significant change was observed over time at both the national and regional levels. Statistical outputs obtained from trend analyses are presented in table 6.

# Table 3. National incidence rates of homicide and suicide among imprisoned people by sex, 2000-2023

|  | **Person-years** | **Number of homicides** | **Homicide incidence rate per 100 000 person-years of imprisonment (95% CI)** | **Number of suicides** | **Suicide incidence rate per 100 000 person-years of imprisonment (95% CI)** |
| --- | --- | --- | --- | --- | --- |
| Male imprisoned population | 926 470 | 921 | 99 (93, 106) | 416 | 45 (41, 49) |
| Female imprisoned population | 75 994 | 4 | 5 (0.11, 10) | 23 | 30 (18, 43) |

# Table 4. National number of deaths by accident and by “other causes” among imprisoned people, 2015-2024

| **Year** | **Number of deaths by accident** | **Number of deaths by “other causes”** |
| --- | --- | --- |
| 2015 | 3 | 2 |
| 2016 | 1 | 5 |
| 2017 | 2 | 7 |
| 2018 | 4 | 9 |
| 2019 | 2 | 9 |
| 2020 | 1 | 7 |
| 2021 | 0 | 6 |
| 2022 | 2 | 8 |
| 2023 | 1 | 15 |
| 2024 | 1 | 17 |
| **TOTAL** | **17** | **85** |

# Table 5. Regional incidence rates of all-cause deaths among imprisoned people

| **Region** | **Person-years** | **Number of all-cause deaths** | **All-cause deaths incidence rate per 100 000 person-years of imprisonment (95% CI)** |
| --- | --- | --- | --- |
| REG. DE ARICA Y PARINACOTA | 32 213 | 77 | 239 (186, 292) |
| REG. DE TARAPACA | 67 533 | 117 | 173 (142, 205) |
| REG. DE ANTOFAGASTA | 48 809 | 118 | 242 (198, 285) |
| REG. DE ATACAMA | 20 231 | 32 | 158 (103, 213) |
| REG. DE COQUIMBO | 44 772 | 117 | 261 (214, 309) |
| REG. DE VALPARAISO | 107 713 | 323 | 300 (267, 332) |
| REG. DEL LIB. OHIGGINS | 54 276 | 152 | 280 (236, 324) |
| REG. DEL MAULE | 51 123 | 129 | 252 (209, 296) |
| REG. DE ÑUBLE | 4 790 | 12 | 251 (109, 392) |
| REG. DEL BIOBIO | 86 415 | 179 | 207 (177, 237) |
| REG. DE LA ARAUCANIA | 45 563 | 101 | 222 (178, 265) |
| REG. DE LOS RIOS | 18 691 | 72 | 385 (296, 474) |
| REG. DE LOS LAGOS | 48 308 | 125 | 259 (213, 304) |
| REG. DE AYSEN | 5 860 | 10 | 171 (65, 2276) |
| REG. DE MAGALLANES Y ANTARTICA | 8 301 | 36 | 434 (292, 575) |
| REG. METROPOLITANA | 379 211 | 1 350 | 356 (337, 375) |
| **TOTAL** | **1 023 809** | **2 950** | **288 (278, 299)** |

Note: Data are reported for the period 2000-2023, only exceptions are Region de Los Ríos, Arica y Parinacota (2008-2023) and Ñuble (2018-2023).

# Table 6. Sensitivity analysis of national suicide Incidence Rate Ratios (IRR) between imprisoned people and the general population: univariable and multivariable region-adjusted Poisson regression models

|  | **Number of observations** | **Coefficient (ß)** | **95% CI** | **p-value** |
| --- | --- | --- | --- | --- |
| **Univariable Poisson regression model** | n=30 |  |  |  |
| **Population group (ref: Imprisoned people)** |  | 3.516 | 3.131-3.948 | <0.0001 |
| **Multivariable Poisson regression model** | n=30 |  |  |  |
| **Population group (ref: Imprisoned people)** |  | 3.565 | 3.174-4.004 | <0.0001 |
| **Regional IRR (ref: Región de Magallanes y Antártica)** |  |  |  |  |
| REG. DE ARICA Y PARINACOTA |  | 0.681 | 0.571-0.811 | <0.0001 |
| REG. DE TARAPACA |  | 0.702 | 0.614-0.804 | <0.0001 |
| REG. DE ANTOFAGASTA |  | 0.699 | 0.619-0.789 | <0.0001 |
| REG. DE ATACAMA |  | 0.846 | 0.741-0.965 | 0.013 |
| REG. DE COQUIMBO |  | 0.795 | 0.708-0.893 | <0.0001 |
| REG. DE VALPARAISO |  | 0.852 | 0.765-0.949 | 0.004 |
| REG. DEL LIB. OHIGGINS |  | 1.010 | 0.904-1.129 | 0.851 |
| REG. DEL MAULE |  | 0.969 | 0.868-1.082 | 0.585 |
| REG. DEL BIOBIO |  | 0.849 | 0.762-0.947 | 0.003 |
| REG. DE LA ARAUCANIA |  | 1.056 | 0.945-1.179 | 0.332 |
| REG. DE LOS RIOS |  | 1.094 | 0.958-1.248 | 0.182 |
| REG. DE LOS LAGOS |  | 1.149 | 1.029-1.284 | 0.014 |
| REG. DE AYSEN |  | 1.178 | 1.008-1.376 | 0.039 |
| REG. METROPOLITANA |  | 0.731 | 0.659-0.811 | <0.0001 |

Abbreviations: IRR: Incidence Rate Ratios; 95% CI: 95% Confidence Intervals. Note: The variable “population group” is dichotomous (1=imprisoned people, 0=general population) and “Regional IRR” is categorical, with “Región de Magallanes y Antártica” as the reference, as it exhibited the highest suicide incidence among imprisoned people. The Ñuble region was excluded from the analysis because the coverage periods between databases (Gendarmería and DEIS) did not match.

# Table 7. National Incidence Rate Ratios between imprisoned people and the general population: A) Both sexes; B) Men; C) Women

A) Incidence Rate Ratios (IRR) in both sexes

|  |  | **Imprisoned people** | | **General population** | |  |  |
| --- | --- | --- | --- | --- | --- | --- | --- |
|  | **Period covered** | **Number of incidents** | **Person-years** | **Number of incidents** | **Person-years** | **IRR** | **95% CI** |
| **Homicide** | 2011-2023 | 610 | 572 432 | 8 932 | 241 425 614 | 29 | 27-31 |
| **Suicide** | 2000-2021 | 378 | 910 109 | 37 003 | 382 030 963 | 4 | 4-5 |
| **External causes of death** | 2011-2021 | 743 | 480 077 | 25 020 | 202 213 743 | 13 | 12-13 |
| **All-cause mortality** | 2000-2023 | 3 024 | 1 002 464 | 2 404 746 | 421 242 834 | 0.53 | 0.51-0.55 |

Abbreviations: IRR: Incidence Rate Ratios; 95% CI: 95% Confidence Intervals.

B) Incidence Rate Ratios (IRR) in men

|  |  | **Imprisoned people** | | **General population** | |  |  |
| --- | --- | --- | --- | --- | --- | --- | --- |
|  | **Period covered** | **Number of incidents** | **Person-years** | **Number of incidents** | **Person-years** | **IRR** | **95% CI** |
| **Homicide** | 2005-2023 | 847 | 772 406 | 11 968 | 170 058 611 | 16 | 15-17 |
| **Suicide** | 2003-2021 | 314 | 751 233 | 32 112 | 166 590 495 | 2 | 2-2 |
| **External causes of death** | 2005-2021 | 1 070 | 687 010 | 38 991 | 150 570 485 | 6 | 6-6 |
| **All-cause mortality** | 2003-2023 | 2 686 | 836 629 | 1 148 455 | 186 078 621 | 0.52 | 0.50-0.54 |

Abbreviations: IRR: Incidence Rate Ratios; 95% CI: 95% Confidence Intervals.

C) Incidence Rate Ratios (IRR) in women

|  |  | **Imprisoned people** | | **General population** | |  |  |
| --- | --- | --- | --- | --- | --- | --- | --- |
|  | **Period covered** | **Number of incidents** | **Person-years** | **Number of incidents** | **Person-years** | **IRR** | **95% CI** |
| **Homicide** | 2011-2023 | 1 | 45 714 | 471 | 121 369 780 | 6 | 0.14-31 |
| **Suicide** | 2003-2021 | 20 | 62 658 | 6 017 | 168 395 051 | 9 | 5-14 |
| **External causes of death** | 2011-2021 | 17 | 38 755 | 3 856 | 101 646 033 | 12 | 7-19 |
| **All-cause mortality** | 2003-2023 | 93 | 69 617 | 1 013 081 | 188 118 798 | 0.25 | 0.20-0.30 |

Abbreviations: IRR: Incidence Rate Ratios; 95% CI: 95% Confidence Intervals.

**Incidence Rate Ratios (IRR) years of reference**

External causes of death included homicides and suicides comparing the imprisoned and general populations. Data for this classification encompassed the period 2011–2021 for both sexes (men: 2005-2021 and women: 2011-2021).

The reference periods used to estimate the IRR presented in the main text were as follows: For all-cause deaths, the analysis covered 2000–2023 for both sexes, and 2003–2023 for men and women separately. For homicides 2011–2023 (men: 2005–2023, and women: 2011–2023), and 2000–2021 for suicides (separated by sex: 2003–2021).

# Table 8. National and regional changes over time in external causes of death (homicide, suicide) among imprisoned people: Crude first-order Prais-Winstein regression outputs

| **National level** | | | | |  |
| --- | --- | --- | --- | --- | --- |
|  | **Period** | **Coefficient (ß)** | **95% CI** | **p-value** | **ρ (rho)** |
| Homicide | 2000-2024 | 2.430 | 0.188, 4.671 | 0.035 | 0.371 |
| Suicide | 2000-2024 | 0.443 | - 0.592, 1.480 | 0.385 | 0.108 |
| **Regional level** | | | | |  |
|  | **Period** | **Coefficient (ß)** | **95% CI** | **p-value** | **ρ (rho)** |
| **REG. DE ARICA Y PARINACOTA** |  |  |  |  |  |
| Homicide | 2008-2023 | 3.554 | -0.886, 7.994 | 0.108 | -0.251 |
| Suicide* | 2008-2023 | - | - | - |  |
| **REG. DE TARAPACA** |  |  |  |  |  |
| Homicide | 2000-2023 | 4.104 | 1.373,6.835 | 0.005 | -0.267 |
| Suicide* | 2000-2023 | - | - | - |  |
| **REG. DE ANTOFAGASTA** |  |  |  |  |  |
| Homicide | 2000-2023 | 2.980 | -0.263, 6.224 | 0.070 | -0.057 |
| Suicide* | 2000-2023 | - | - | - |  |
| **REG. DE ATACAMA** |  |  |  |  |  |
| Homicide* | 2000-2023 | - | - | - |  |
| Suicide* | 2000-2023 | - | - | - |  |
| **REG. DE COQUIMBO** |  |  |  |  |  |
| Homicide | 2000-2023 | 4.101 | 0.463, 7.739 | 0.029 | -0.218 |
| Suicide | 2000-2023 | -1.229 | -4.816, 2.357 | 0.485 | -0.238 |
| **REG. DE VALPARAISO** |  |  |  |  |  |
| Homicide | 2000-2023 | 5.867 | 1.401, 10.333 | 0.012 | 0.451 |
| Suicide | 2000-2023 | 0.423 | -1.924, 2.770 | 0.712 | 0.254 |
| **REG. DEL LIB. OHIGGINS** |  |  |  |  |  |
| Homicide | 2000-2023 | 7.463 | 2.371, 12.554 | 0.006 | 0.075 |
| Suicide | 2000-2023 | 0.774 | -4.167, 5.716 | 0.748 | -0.027 |
| **REG. DEL MAULE** |  |  |  |  |  |
| Homicide | 2000-2023 | 0.743 | -2.237, 3.725 | 0.610 | -0.162 |
| Suicide | 2000-2023 | 0.538 | -2.115, 3.192 | 0.678 | -0.139 |
| **REG. DE ÑUBLE** |  |  |  |  |  |
| Homicide* | 2018-2023 | - | - | - |  |
| Suicide* | 2018-2023 | - | - | - |  |
| **REG. DEL BIOBIO** |  |  |  |  |  |
| Homicide | 2000-2023 | 1.283 | -2.233, 4.800 | 0.457 | 0.077 |
| Suicide | 2000-2023 | 1.953 | -0.877, 4.785 | 0.166 | 0.458 |
| **REG. DE LA ARAUCANIA** |  |  |  |  |  |
| Homicide | 2000-2023 | 5.319 | 1.554, 9.084 | 0.008 | -0.054 |
| Suicide | 2000-2023 | -0.670 | -3.484, 2.144 | 0.626 | 0.004 |
| **REG. DE LOS RIOS** |  |  |  |  |  |
| Homicide | 2008-2023 | 21.576 | 11.389, 31.762 | <0.0001 | -0.019 |
| Suicide | 2008-2023 | 5.012 | -1.196, 11.220 | 0.105 | -0.230 |
| **REG. DE LOS LAGOS** |  |  |  |  |  |
| Homicide | 2000-2023 | 4.842 | 0.567, 9.117 | 0.028 | 0.165 |
| Suicide | 2000-2023 | 4.230 | -0.393, 8.855 | 0.071 | -0.221 |
| **REG. DE AYSEN** |  |  |  |  |  |
| Homicide* | 2000-2023 | - | - | - |  |
| Suicide* | 2000-2023 | - | - | - |  |
| **REG. DE MAGALLANES Y ANTARTICA** |  |  |  |  |  |
| Homicide* | 2000-2023 | - | - | - |  |
| Suicide | 2000-2023 | 7.745 | -1.824, 17.314 | 0.107 | -0.412 |
| **REG. METROPOLITANA** |  |  |  |  |  |
| Homicide | 2000-2023 | 1.291 | -4.111, 6.694 | 0.625 | 0.400 |
| Suicide | 2000-2023 | 0.351 | -0.726, 1.428 | 0.506 | -0.132 |

Note: ρ (rho) ≠ 0 indicates autocorrelation. *Regions reporting fewer than 15 incidents for the complete period were excluded from analysis.

# Table 9. National and regional trends in homicide among imprisoned people: First-order Prais-Winstein regression outputs adjusted for trend break and the COVID-19 pandemic effects

| **National level** | | | | |  |
| --- | --- | --- | --- | --- | --- |
|  | **Period** | **Coefficient (ß)** | **95% CI** | **p-value** | **ρ (rho)** |
| **Univariable model** | 2000-2014 |  |  |  | -0.258 |
| Time (years) |  | 5.366 | 3.064, 7.667 | <0.0001 |  |
| **Multivariable model** | 2000-2024 |  |  |  | -0.194 |
| Time (years) |  | 5.438 | 3.443, 7.433 | <0.0001 |  |
| Trend break (years ≥ 2015) |  | 190.985 | 115.407, 266.562 | <0.0001 |  |
| Interaction term (time * trend break) |  | -12.267 | -16.519, -8.014 | <0.0001 |  |
| Pandemic effect (years 2020-2021) |  | 47.267 | 18.444, 76.089 | 0.003 |  |
| **Regional level** | | | | |  |
|  | **Period** | **Coefficient (ß)** | **95% CI** | **p-value** | **ρ (rho)** |
| **Multivariable model** | 2005-2023 |  |  |  | -0.300 |
| **REG. METROPOLITANA** |  |  |  |  |  |
| Time (years) |  | -5.804 | -8.888, -2.721 | 0.001 |  |
| Pandemic effect (year 2020) |  | 125.303 | 32.989, 217.617 | 0.011 |  |
| **Multivariable model** |  |  |  |  | -0.083 |
| **REG. DE LOS RIOS** | 2008-2023 |  |  |  |  |
| Time (years) |  | 19.284 | 8.516, 30.052 | 0.002 |  |
| Pandemic effect (year 2020-2021) |  | 79.345 | -75.121, 233.812 | 0.287 |  |

Note: ρ (rho) ≠ 0 indicates autocorrelation.

# Table 10. Principal Component Analysis (PCA) eigenvalues

| **Component** | **Eigenvalue** | **Variance explained (%)** | **Cumulative variance explained (%)** |
| --- | --- | --- | --- |
| Component 1 | 2.195 | 54.9 | 54.9 |
| Component 2 | 1.173 | 29.3 | 84.2 |
| Component 3 | 0.539 | 13.4 | 97.7 |
| Component 4 | 0.091 | 2.2 | 100 |

# Table 11. PCA factor loadings

| **Variable** | **Component 1** | **Component 2** | **Component 3** | **Component 4** |
| --- | --- | --- | --- | --- |
| Proportion of people convicted of homicide | 0.639 | -0.109 | -2.280 | 0.707 |
| Proportion of people convicted of sexual crimes | 0.531 | 0.059 | 0.833 | -0.140 |
| Occupancy | -0.282 | 0.817 | 0.199 | 0.461 |
| Proportion of non-national prisoners | -0.478 | -0.562 | 0.432 | 0.517 |

# Table 12. Linear regression R-squared (R²) for external causes of death (homicide, suicide) and suicide incidence with PCA Component 1 “Imprisoned nationals convicted of homicide or sexual offences” as the independent variable

|  |  | **R²** | **Adjusted R²** |
| --- | --- | --- | --- |
| External causes of death | Univariable model with Comp. 1 | 0.3978 | 0.3548 |
|  | Multivariable model with Comp. 1 adjusted for Comp. 2 | 0.4109 | 0.3203 |
| Suicide | Univariable model with Comp. 1 | 0.4188 | 0.3773 |
|  | Multivariable model with Comp. 1 adjusted for Comp. 2 | 0.4244 | 0.3358 |

Note: Comp. 1: “Imprisoned nationals convicted of homicide or sexual offences”; Comp. 2: “Imprisoned nationals in overcrowded settings”.

# Table 13. Component 1: “Imprisoned nationals convicted of homicide or sexual offences” scores by region

| **Region** | **Component 1 score** |
| --- | --- |
| ARICA Y PARINACOTA | -1.59 |
| TARAPACÁ | **-2.32** |
| ANTOFAGASTA | -1.90 |
| ATACAMA | -1.85 |
| COQUIMBO | 0.41 |
| VALPARAÍSO | -0.69 |
| REGION METROPOLITANA | -0.60 |
| LIB. B. O’ HIGGINS | 0.31 |
| DEL MAULE | -0.59 |
| ÑUBLE | -0.34 |
| BIOBÍO | 0.74 |
| LA ARAUCANÍA | 0.68 |
| LOS RÍOS | **1.97** |
| LOS LAGOS | 1.62 |
| AYSÉN | **2.54** |
| MAGALLANES Y ANTÁRTICA | 1.58 |

Note: High scores indicate a greater presence of high-risk national prisoners within penal facilities; a profile associated with increased suicide incidence. Tarapacá presented the lowest score. Aysén and Los Ríos reached the highest scores.
